# Temporal gradient analysis of blood glucose responses to non-standard physical activity: a free-living study in type 1 diabetes

**DOI:** 10.3389/fspor.2026.1718510

**Published:** 2026-02-13

**Authors:** Ahmad Bilal, Hood Thabit, Paul W. Nutter, Simon Harper

**Affiliations:** 1 Department of Computer Science, The University of Manchester, Manchester, United Kingdom; 2 Diabetes, Endocrine and Metabolism Centre, Manchester Royal Infirmary, Manchester University NHS, Manchester, United Kingdom; 3 Division of Diabetes, Endocrinology and Gastroenterology, School of Medical Sciences, The University of Manchester, Manchester, United Kingdom

**Keywords:** type 1 diabetes, physical activity, blood glucose, continuous glucose monitoring, wearable sensors, gradient analysis, personalised modelling, free-living

## Abstract

**Introduction:**

Daily physical activity (PA) impacts blood glucose (BG) in individuals with Type 1 Diabetes Mellitus (T1DM), with effects varying by intensity, duration, and timing. Predicting BG changes during free-living activity remains challenging but may help prevent hypoglycaemia. Previous studies have focused on the impact of PA on BG levels, but only during exercise sessions, not throughout the entire day.

**Methods:**

Using retrospective data from eight individuals with T1DM (mean age 67 years; 3 female, 5 male), we analysed whether non-standard PA, defined as activity exceeding the individual’s mean habitual level in a preceding interval, was associated with steeper downward trends in BG. PA was quantified using wrist-worn accelerometry, and BG responses were analysed using gradient-based methods across 20, 40, and 60 min time windows.

**Results:**

Two hypotheses were evaluated. Hypothesis 1 assessed whether BG decline intensified during existing downward trends and achieved an accuracy above 83.33%, with F1-scores exceeding 0.83 at shorter intervals. Hypothesis 2 examined BG declines following prior increases and showed greater variability; accuracy ranged from 73.53% to 88.33%, with the lowest F1-score of 0.75 at the 60 min window.

**Conclusion:**

We have found a reliable correlation between increased levels of PA and BG levels under free-living conditions. These findings establish a foundation for future work aimed at quantifying BG responses to PA and developing personalised decision-support tools for insulin or carbohydrate adjustment.

## 1 Introduction

Hypoglycaemia induced by physical activity (PA), often termed activity-induced hypoglycaemia, is a significant concern for individuals with type 1 diabetes mellitus (T1DM) and those involved in strenuous exercise [1]. Understanding the intricate relationship between PA intensity and blood glucose (BG) is crucial for mitigating the risks associated with hypoglycaemia during and after PA. This understanding is vital for tailoring strategies to maintain stable BG levels in active individuals [2]. Professional organisations and international consensus recommend that individuals with T1DM maintain their BG levels within 3.9–10 mmol/L [3, 4]. PA undertaken daily is one of the most crucial factors influencing BG levels and requires management [1].

PA can induce hypoglycaemia in individuals with T1DM due to increased BG utilisation and insulin sensitivity, potentially leading to delayed onset if not appropriately managed [5]. The intensity and duration of PA can significantly impact BG levels, particularly for individuals with T1DM [6]. Recognising the diverse range of PAs is crucial while exploring the interplay between PA and BG levels. This extends beyond strenuous exercise, encompassing everyday PA such as walking, gardening, climbing stairs, and household chores. Even routine PA, from a simple supermarket visit to running a marathon, can intricately influence BG levels.

Our research diverges from studies focused on time-based exercises scheduled to begin at particular times each day, such as time-oriented workout routines, for predicting BG levels. What sets our study apart is its emphasis on PA at any given time, incorporating various everyday tasks unrelated to exercise. Unlike existing research, our focus extends beyond scheduled exercises, comprehensively exploring the impact of diverse and spontaneous PA on BG. While low-level PA, like light walking, may have minimal immediate effects, regular engagement can improve insulin sensitivity over time [7, 8]. Moderate-level PA can cause a decrease in BG levels during the PA [9]. High-level PA leads to a lower initial decline in BG during the PA. Still, they are linked to more sustained reductions in post-exercise BG levels compared to moderate-level PA [10]. Our research examines a wide range of PA, including strenuous exercises.

Activity wearables, or fitness trackers, are essential tools for monitoring PA and health metrics, particularly in managing T1DM [11]. They capture parameters like step count, distance, active minutes, sedentary periods, and heart rate, offering insights into PA intensity and its impact on BG levels [12, 13]. Integrating data from these devices into diabetes management platforms enables personalised insulin dosing and supports broader management strategies [14]. Smartphone applications enhanced by Machine Learning (ML) further allow users to log meals, activity, stress, and sleep, helping forecast BG fluctuations [15]. Increasingly, health platforms are combining these data streams to deliver a comprehensive, data-driven approach to diabetes self-management [16].

T1DM prediction can be studied through physiological, data-driven, and hybrid models [17]. Physiological models involve mathematical formulas to simulate the dynamics of BG, insulin, and meals, whereas data-driven models use historical data and can be time series, ML, or hybrid models; hybrid models combine both approaches to enhance prediction accuracy [17]. This study adopts a data-driven approach, specifically emphasising statistical methods, for BG prediction while incorporating individual self-recorded historical data, known for its black box nature due to reduced reliance on detailed physiological understanding. The data-driven framework considers various models, including time series and statistical methods, with a specific focus on assessing the impact of PA on BG dynamics. This choice is made to leverage the adaptability of data-driven models in capturing the nuanced relationships between PA patterns and BG levels, aiming for accurate predictions and tailored insights [17].

Identifying physical exercise promptly among individuals with T1DM can facilitate timely modifications in therapy to mitigate the risk of hypoglycaemia [18]. In this context, the classification of PA intensity levels becomes crucial. Heart rate is another important parameter in predicting BG levels, with algorithms incorporating heart rate data demonstrating efficacy in forecasting minimum BG levels and hypoglycaemia following structured exercise regimens [19]. However, extending these predictive capabilities to daily living remains challenging due to the dynamic and varied intensity levels encountered throughout a typical day.

Predicting BG fluctuations in people with T1DM is complex due to multiple influencing factors, including past carbohydrate intake, insulin dosage, PA, and stress levels [43]. Overcoming individual variability, data integration, dynamic physiological regulation, interpretability, and validation challenges is critical for developing practical predictive tools [20]. Creating BG prediction models that account for PA in real-world settings holds considerable promise for T1DM management. Such models can provide valuable insights into how different types and intensities of PA impact BG levels, supporting strategies for hypoglycaemia prevention and enabling more precise and individualised glycaemic control.

Artificial Intelligence (AI) and ML models have demonstrated promise in predicting future BG levels based on various inputs, including PA [21–24]. However, most existing algorithms focus on structured exercise rather than encompassing the full spectrum of daily activities [25]. This distinction is critical, as everyday PA involves a wider range of intensities and durations, making BG prediction more challenging and requiring more nuanced algorithmic approaches [26]. Moreover, BG regulation is influenced by multiple interacting factors, including diet, insulin administration, and physiological variability [27]. Unexpected changes such as irregular meals, insulin fluctuations, or unplanned PA can introduce significant variability into ML inputs, potentially reducing predictive accuracy. These complexities highlight the need to assess PA effects in real-world settings. Our aim is to quantify PA-induced glycaemic demand and establish equivalences between PA levels, insulin dosages, and carbohydrate adjustments.

While prior studies have examined spontaneous PA in T1D, most have focused on overall PA levels or structured exercise. One study contrasted high vs. low exercise in free-living adults but did not evaluate episode-specific BG responses [28]. Another highlighted the clinical challenges of planned vs. spontaneous PA, but provided conceptual rather than quantitative frameworks [29]. More recently, work in children explored the acute effects of spontaneous and structured PA, emphasising activity timing, carbohydrate intake, and insulin on board [30]. In contrast, our study examines episode-level responses in older adults with long-standing T1DM, defining non-standard PA relative to each individual’s habitual baseline and assessing its short-term impact on BG gradients.

It is well established through clinical observation and empirical studies that daily insulin requirements in individuals with T1DM are influenced by their levels of habitual PA [6, 31]. Basal insulin doses, in particular, are often adjusted based on an individual’s average daily energy expenditure. However, PAs that significantly exceed this average, such as spontaneous high-intensity PA or unusually long bouts of exercise, require special attention due to their variable and sometimes unpredictable effects on BG levels. To systematically address this, we categorised PA data into two groups: activities exceeding the individual’s mean daily PA were labelled “non-standard PA,” while those below this level were classified as “standard PA.” Standard PA typically includes habitual activities with lower variability, such as commuting or household tasks, whereas non-standard PA encompasses deviations from routine, often characterised by increased intensity or duration.

The vision of this work is to develop an adaptive algorithm that integrates daily PA patterns into insulin dosing and carbohydrate intake adjustments, enabling real-time modifications in decision-support tools such as bolus calculators [32]. By analysing the impact of low, medium, and high PA levels, including structured exercise, on BG, the algorithm aims to dynamically optimise insulin and carbohydrate recommendations to enhance glycaemic control. The goal is to establish a clinically relevant equivalence between PA levels and insulin dosage, allowing individuals with T1DM to manage BG fluctuations more effectively under free-living conditions.

In this context, we introduce the concept of *non-standard physical activity*, defined as PA that exceeds an individual’s mean habitual daily PA level within a preceding time interval. This personalised definition based on deviations has not been explicitly operationalised in prior T1DM literature, where PA is typically categorised using fixed intensity thresholds or structured exercise sessions. Our definition is therefore intentionally individual-specific, reflecting inter-individual variability in baseline activity patterns and physiological responses. Accordingly, no universal minimum intensity or duration threshold is imposed. Instead, non-standard PA is identified through deviations above each participant’s own baseline (mean PA), which is calibrated over 20, 40, and 60 min windows. Such deviations correspond to activity levels that are not fully compensated by basal insulin alone and therefore pose an increased risk of glycaemic instability. This approach enables the capture of spontaneous, unplanned activities that are of higher intensity or longer duration relative to an individual’s typical daily behaviour and that may meaningfully influence glycaemic dynamics.

In future applications, this framework could be integrated into personalised decision-support systems, including adaptive bolus calculators or open-loop insulin delivery systems. Specifically, the algorithm could (i) generate alerts when non-standard PA coincides with a predicted decline in BG, (ii) support preventive carbohydrate recommendations to mitigate the risk of imminent hypoglycaemia, and (iii) inform insulin dosing adjustments by accounting for PA-related changes in insulin sensitivity and BG dynamics. As such, this work represents an initial, proof-of-concept step toward developing PA-aware diabetes management systems that better reflect real-world, free-living behaviour.

Here we examine the different levels of PA and their corresponding impact on BG. Research closely aligned with our current investigation has been carried out, such as a BG model based on support vector regression achieving 94% sensitivity in predicting nocturnal hypoglycaemic events [33]. Another study indicated that more than 70% of nocturnal hypoglycaemia might be avoided with predictions performed by support vector machines, achieving a sensitivity and specificity of 78.75% and 82.15%, respectively [34]. The primary distinction between their approach and ours lies in the timing of our predictions for BG levels. We focus on daytime predictions, considering the influence of PA on BG levels throughout the day, whereas in [33], the emphasis is on nighttime predictions.

Our methodology differs from other studies, as we do not incorporate food intake and insulin dosages while concentrating solely on utilising PA data. We deliberately focused only on the effect of PA on BG, as in real-world practice, dietary information is often missing or incorrectly entered. In a daily routine, people tend to bolus rather than consistently input meal information. Our aim was to ensure that the work reflects real-world behaviour and can be applied without requiring additional dietary input, making the approach more practical.

Open-loop diabetes technologies struggle with spontaneous, real-world PA, which can boost BG utilisation and insulin sensitivity during and after PA. We aim to develop a PA-aware algorithm that realigns carbohydrate and insulin recommendations, complementing bolus calculators and decision-support systems. Rather than focusing solely on structured exercise, we investigate free-living, day-long PA, including spontaneous bouts linked to glycaemic instability. In this paper, we define non-standard PA as activity exceeding an individual’s average habitual level in a prior interval. Using gradient analysis over 20, 40, and 60 min windows, we assess whether (i) a declining BG experiences a steeper drop following non-standard PA, and (ii) a rising BG sees a subsequent decline.

## 2 Methodology

This study analyses the temporal relationship between non-standard PA and subsequent BG trends using a gradient-based framework that measures change of BG (ΔBG/Δt) [35]. Specifically, we evaluate whether the occurrence of non-standard PA is associated with a steeper decline in BG in the immediately following time interval compared to the preceding interval. The analytical framework focuses on detecting changes in the direction and rate of BG trends rather than estimating the absolute magnitude of BG decline attributable to PA. Quantifying the size of BG changes associated with different types or intensities of non-standard PA will be addressed in future work. Here, the emphasis is on identifying whether non-standard PA events are temporally associated with increased glycaemic instability, as reflected by steeper BG gradients.

### 2.1 Participants

A secondary analysis was performed using data from a cohort of eight individuals with T1DM who participated in a clinical study [36] when they were using open-loop (OL) insulin delivery systems. In the parent study, eligibility criteria included age ≥ 60 years and T1DM for at least one year. Key exclusion criteria comprised non-T1DM; recent closed-loop system use; physical or psychological conditions likely to interfere with study conduct or interpretation; use of BG-lowering agents other than insulin; untreated disease, adrenal insufficiency, or hypothyroidism; recurrent severe hypoglycaemia; extreme daily insulin dose thresholds; and severe visual or hearing impairment. Collectively, these criteria were designed to eliminate the influence of T1DM-related comorbidities or concurrent treatments on glycaemic outcomes [36].

Table 1 summarises the demographics and clinical characteristics of the eight included participants (all aged 60 years or older), who were engaged in routine daily activities under free-living conditions. For this secondary analysis, we further included only individuals with complete and temporally aligned PA collected from Philips Actiwatch and continuous glucose monitoring, which was Dexcom G6 data, for at least five consecutive days. Participants were excluded if PA or CGM records were incomplete, misaligned, or missing during the observation window. Applying these data-availability criteria yielded the eight individuals analysed here.

**Table 1:** Participant demographics and clinical characteristics.

Participant	Baseline HbA1c	Gender	Age at recruitment	Total daily dose of insulin	Total basal insulin dose	Weight	Height	BMI	Duration of diabetes	Duration of pump use
ID	(mmol/mol)	(M = 0, F = 1)	(years)	(U)	(U)	(kg)	(m)	(kg/m^2^)	(years)	(years)
ID01	51	1	64.6	59.0	28.3	75.0	1.65	27.7	44.7	10.5
ID02	43	0	70.4	27.8	10.6	68.0	1.74	22.6	53.1	8.3
ID03	54	1	60.9	24.9	8.1	58.7	1.59	23.2	38.6	11.7
ID04	76	0	80.0	45.0	26.4	101.8	1.71	34.8	47.9	10.9
ID05	66	0	70.0	39.3	17.5	80.0	1.74	26.4	35.4	30.4
ID06	50	0	60.0	49.4	30.0	90.0	1.83	26.9	37.6	18.7
ID07	71	0	67.6	51.0	29.3	96.0	1.84	28.4	35.1	19.0
ID08	57	1	62.5	84.0	28.0	73.4	1.60	28.7	34.6	7.6

HbA1c, glycated haemoglobin (reported in mmol/mol); BMI, body mass index; U, units; M, male; F, female.

All participants used Dexcom G6 continuous glucose monitoring and Dana Diabecare RS insulin pumps during the study.

Although the number of participants was small, each contributed between 5 and 8 consecutive days of high-density data, yielding approximately 28,800–46,000 PA epochs (15 s resolution) and 1,440–2,300 BG readings (5 min resolution) per participant. This richness of within-person data supports hypothesis-driven temporal analysis under free-living conditions. While detailed participant-level comorbidity variables beyond the parent study eligibility screening were not available in the extracted dataset, the strict exclusion criteria of the parent trial eliminated the likelihood that major comorbidities independently influenced the observed glycaemic responses.

The assumption that adaptive systems require large user populations reflects a misunderstanding of how personalisation operates in longitudinal, within-participant settings. Unlike population-level models that derive inferential power primarily from between-participant variability, adaptive and personalised systems accumulate evidence over time from repeated observations within individual users. Consequently, the analytical focus shifts from cross-sectional population inference to within-participant pattern consistency and temporal reliability.

In the present framework, each analysed time window constitutes an observation, and each participant contributes hundreds to thousands of repeated PA–BG interaction events across multiple days. Statistical sensitivity is therefore driven by the density, consistency, and directionality of within-participant observations, rather than by the absolute number of participants. This approach is particularly appropriate in physiological modelling, where responses to physical activity often exhibit lower within-person variability than between-person variability.

Accordingly, no a priori power analysis was performed, as such analyses are designed to inform sample size requirements prior to data collection under population-level assumptions. Instead, given that data collection was complete, we assessed whether the observed classification performance across participants could be attributed to random variation. Therefore, post hoc non-parametric statistical analyses were performed to evaluate the strength and consistency of the observed signal across individuals, while preserving the personalised structure of the data. These analyses are reported in the Results section. The present study is therefore intended as a proof-of-concept evaluation of an adaptive, PA-aware BG modelling framework, demonstrating robust within-participant signal consistency under free-living conditions rather than population-level generalisation.

### 2.2 Materials

Continuous Glucose Monitoring (CGM): Participants utilised Dexcom G6 to monitor BG levels (mmol/L), with data collected at 5 min intervals throughout the study.

Insulin Administration: The Dana Diabecare RS insulin pump is used for insulin administration.

Activity Monitoring: PA was assessed using the Philips Actiwatch. The device records an “Activity Count” metric derived from peak acceleration detected within each 15 s epoch and is widely used for activity and sleep–wake pattern analysis [37]. In accordance with the parent study protocol, the Actiwatch was worn on the wrist of the non-dominant hand.

Importantly, this study does not rely on absolute activity intensity thresholds or estimates of whole-body movement, and no manufacturer-specific wrist-normalisation algorithm was applied. Instead, PA is analysed relative to each participant’s own habitual baseline, with non-standard PA defined as deviations above the individual’s mean activity level within 20, 40, and 60 min windows. As a result, potential differences arising from wrist placement or movement amplitude do not materially affect the analysis, as the framework focuses on within-individual deviations rather than absolute intensity classification.

### 2.3 Procedure

We selected five consecutive days of participant data to ensure data consistency and relevance. Our initial step involved gathering data on both PA and BG levels. This study quantified PA using activity counts collected from the Philips Actiwatch device. The Actiwatch measures activity levels by recording wrist movements, providing an objective count of PA that reflects both the intensity and frequency of movement throughout the day. This method is reliable for monitoring daily PA levels without relying on self-reported data, which can often be subjective and inaccurate [38].

Meal and insulin records were intentionally excluded because, in real-life scenarios, dietary entries are often missing or incorrect. In contrast, PA and CGM data are objective and consistently available. By focusing exclusively on PA as the input, we aimed to quantify its independent effect on BG levels and to demonstrate the feasibility of prediction models that do not depend on potentially incomplete meal data. This design reflects real-world usability, where PA and CGM are typically the most reliable sources of data. Furthermore, to aid in interpretation by clinicians, we summarise the logic in a structured pseudocode format Table 2, highlighting key steps from PA aggregation to hypothesis-driven classification of BG trends. This complements the technical descriptions and supports a broader interdisciplinary understanding.

**Table 2:** Structured pseudocode describing the steps involved in predictive activity modelling of glucose dynamics. The framework integrates wearable-derived physical activity (PA) data and continuous glucose monitoring (CGM)-derived blood glucose (BG) data to identify activity-driven glycaemic trends under two hypothesised patterns: (1) accelerated glucose decline during high PA, and (2) delayed glucose decline following an initial rise post-activity.

Physical activity associated glucose dynamics
**Input:** Wearable-derived PA data and CGM-derived BG data
**Output:** Detection of activity-driven glucose trends (accelerated or delayed drops)
**1.** **Activity Aggregation:**
For each interval, assign:
Active if label contains “ACTIVE” Rest if label contains neither “ACTIVE” nor noise indicators **Exclude** if label contains “REST-S” or “EXCLUDED”
**2.** **Interval Resampling and Variability:**
Resample data into 20, 40, or 60 min intervals and compute:
Activity variability = standard deviation of activity Glucose variability = standard deviation of BG values
**3.** **Merge Activity and Glucose Streams:**
Join datasets by timestamp, creating unified records with:
PA label, activity std, glucose std
**4.** **Gradient Calculation:**
For each day between 06:00–23:59, compute:
ActivityGradient = change in activity/time GlucoseGradient = change in BG/time
**5.** **Hypothesis 1 – Accelerated Drop:**
If glucose gradient is falling and continues to fall with steeper slope during high PA:
Classify event as True Positive/False Positive/True Negative/False Negative
**6.** **Hypothesis 2 – Drop After Initial Rise:**
If glucose gradient initially rises and then falls post-activity (above mean–threshold):
Again classify into TP/FP/TN/FN

TP, true positive; FP, false positive; TN, true negative; FN, false negative.

Data cleaning procedures, including the removal or correction of missing and inconsistent records, have been shown to improve classification performance [39]. Therefore, the collected data was cleaned to eliminate inconsistencies or outliers. We observed inconsistencies in the data across different timeframes, including missing BG measurements for certain *x*-minute intervals, even though PA data was available or vice versa. To ensure the accuracy and reliability of our results, we excluded these intervals from our analysis. We prioritised relevant PA data during file parsing, focusing on the individual’s active or resting states. “REST-S” denoted sleep, and “EXCLUDED” data was omitted. Our filtration aimed to exclusively include active and rest periods for subsequent pattern analysis, as illustrated in Algorithm 1. All the algorithms are listed in the Appendix. Our study aims to investigate the impact of PA on BG, focusing specifically on data during active or rest states, excluding sleep, hence why rest and sleep data were omitted.

We know from prior work [40] that resampling improves temporal consistency and reduces noise, providing a basis for time series analysis. Accordingly, we resampled the collected PA and BG data at 20, 40, and 60 min intervals. This approach is supported by studies [6, 41] indicating that PA begins to affect BG approximately 15–20 min after onset, depending on intensity, and its impact can persist for up to 18–24 h. We used 15 s activity counts and 5 min BG (mmol/L) readings as the base units, which were aggregated into 20 min intervals as described in Algorithm 2. This reflects the degree of fluctuation within each window rather than absolute levels. This choice was made to quantify the variability and temporal instability of both signals, which are highly relevant in real-world, free-living conditions where data noise and spontaneous behaviours are common. This resampling enabled the examination of trends and variability in PA and BG over multiple timescales. Notably, the model accounts for individual variability in PA intensity, using activity count as a personalised measure of exertion. This personalised framework supports a more nuanced understanding of how PA intensity influences BG dynamics across individuals.

Resampling was performed using the standard deviation within each time window rather than mean aggregation or point sampling. Physical activity (PA) values recorded at 15 s resolution and blood glucose (BG) values recorded at 5 min resolution were aggregated by computing the standard deviation within 20, 40, and 60 min windows. Using standard deviation allowed us to quantify short-term variability and instability within each window, which is more physiologically meaningful than absolute levels when assessing rapid BG responses to non-standard physical activity under free-living conditions. Each 20 min window summarised up to 80 PA samples (15 s epochs) and four BG samples (5 min readings), capturing within-window fluctuations while reducing the influence of sensor noise, timing misalignment, and delayed physiological responses. This variability-based representation provides a robust foundation for subsequent gradient-based analysis, as all interpretations refer to changes in variability rather than fixed PA or BG values. Representative examples of raw PA inputs and their corresponding resampled outputs are shown in Tables 3, 4.

**Table 3:** Resampled PA data before cleaning and aggregation. Activity counts recorded at 15 s resolution within each 20 min window are summarised using the standard deviation (STD), capturing within-window variability rather than absolute activity levels. Interval status labels within each window are consolidated using a rule-based approach: if any epoch is labelled as ACTIVE, the entire window is classified as ACTIVE; windows containing REST-S (sleep) or EXCLUDED labels are discarded; all remaining windows are classified as REST. This procedure ensures that retained windows (ACTIVE and REST) represent either active or resting free-living behaviour while excluding sleep and invalid data.

Input		
Timestamp	Activity count	Interval status
10:00:15	18	REST
10:00:30	45	ACTIVE
10:00:45	62	ACTIVE
10:01:00	38	REST
.	.	.
.	.	.
10:20:00	0	ACTIVE

**Table 4:** Resampled PA data after cleaning and aggregation. Activity counts within the 20 min window are summarised using standard deviation (STD). The interval is labelled as ACTIVE because as last one ACTIVE epoch occurred within the window.

Output		
Timestamp	Activity	Interval status
10:20:00	22.4	ACTIVE

The next step involves combining the cleaned and resampled datasets for the analysis. The Algorithm 3 outlines the process of merging the PA and BG data based on the shared “Timestamp” column. It is important to note that timestamp alignment was feasible because the BG data had already been resampled at 20 min intervals, and the PA data, originally recorded every 15 s, was also resampled to 20 min intervals. This ensured that both datasets shared consistent time bins, enabling a direct one-to-one merge. After merging, rows containing missing values were excluded. The resulting dataset, stored in the variable bloodglucose, contains synchronised PA and BG data, which is now ready for further analysis.

The importance of the gradient in time series data analysis lies in its ability to quantify the rate of change over time, providing valuable insights into temporal dynamics [42]. This significance becomes evident when examining the impact of PA on BG levels, where the gradient serves as a crucial metric for understanding the quantitative relationship between different PA patterns and BG fluctuations. In support of our investigation into the correlation between PA and BG, we analysed this data by determining the gradient of the PA and BG data over 20, 40 and 60 min timeframes. We continuously performed gradient analysis on all five days for each individual to identify significant increases in PA and corresponding decreases in BG levels. This process was crucial for establishing the groundwork for our analysis. We conducted this analysis daily for all eight participants, ensuring thorough examination across the entire participant group.

The Algorithm 4 calculates the daily gradients for both PA and BG levels. For each day, the rate of change in PA and BG between consecutive timestamps is computed. These gradients are derived by calculating the differences in PA and BG values over the time difference between consecutive data points. The resulting gradients are then averaged over all days, providing a measure of the overall rate of change in both PA and BG levels. The final gradients are stored in a data frame, gradient_std, which contains both the PA and BG gradients for each timestamp in the dataset.

### 2.4 Data analysis

**Figure 1:**
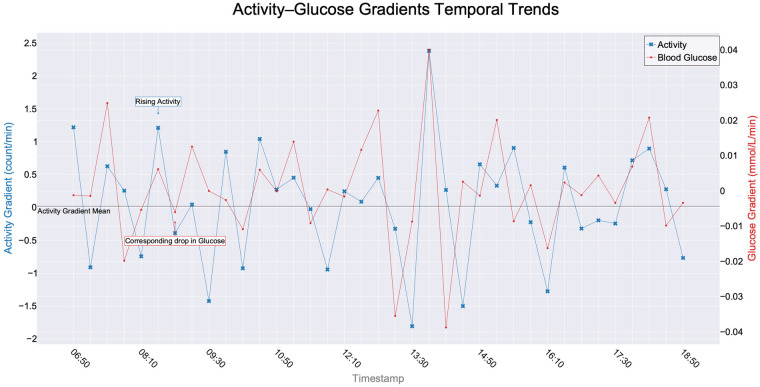
Temporal interplay for real participant (ID05): A gradient analysis illustrating the correlation between an individual’s PA and BG levels (based on calculated gradients) throughout one representative day. The data, recorded at 20 min intervals, captures the dynamic relationship between PA and BG fluctuations. Gradients can be positive or negative, indicating increasing or decreasing trends. Units are as follows: PA gradient is expressed in activity counts per minute, and BG gradient is expressed in mmol/L per min, with the time interval explicitly defined by the 20 min resampling. This figure does not represent simulated data or an average across participants. Here, the mean activity gradient = 0.0191 counts/min.

When examining the impact of PA on BG levels, Figure 1 illustrates the combined gradient analysis of an individual’s PA and BG levels over a day for a specific period. The data points are spaced at 20 min intervals. When the gradient of BG is observed to be decreasing at time *x*, coinciding with a non-standard PA, a more pronounced decline in the BG gradient is observed after x+20 min compared to x−20 min. This means that a corresponding decline in the BG gradient is observed for every rising non-standard PA. Studies indicate that a decrease in BG is attributed to various factors, with PA being one of the contributing elements [43].

In this study, we analyse the Activity Gradient Mean, which represents the PA change rate over time and is expressed in activity counts per minute. This means that, unlike raw PA data, the gradient can take both positive and negative values, depending on whether PA levels increase or decrease at a given moment. Similarly, the BG gradient quantifies changes in BG concentration over time and is expressed in mmol/L per min. These gradient values can be either positive (indicating an increase) or negative (indicating a decrease), depending on the change direction over each interval. This approach enables us to capture temporal dynamics rather than relying on raw values alone. If at time x, the BG concentration is 7.0 mmol/L, and at x+20 min, it decreases to 6.5 mmol/L, the gradient is calculated using the formula:

$$\text{Gradient}=\frac{\text{Glucose at}\;(x+t)-\text{Glucose at}\;x}{t}$$

Substituting the values:

$$\text{Gradient}=\frac{6.5-7.0}{20}=-0.025\;\text{mmol/L per min}$$

This results in a negative gradient, indicating a decrease in BG concentration during the 20 min interval. The units used in our analysis remain consistent with this approach: counts for PA and mmol/L for BG concentration, while the time component (per second, per minute, or per hour) is inherently defined by the timeframe of gradient computation.

Subsequently, we investigated the occurrence of non-standard PA within the 20 min timeframe preceding the observed decline. This phenomenon formed the basis of our initial hypothesis Algorithm 5, where we hypothesise that a further decline in the BG gradient exists after a specific duration when the BG gradient is already falling, accompanied by non-standard PA. This indicates that the decrease in BG tends to intensify in the presence of a non-standard PA. When the participant stops non-standard PA or consumes fewer carbohydrates, it stabilises the BG gradient or eventually causes it to rise.

**Figure 2:**
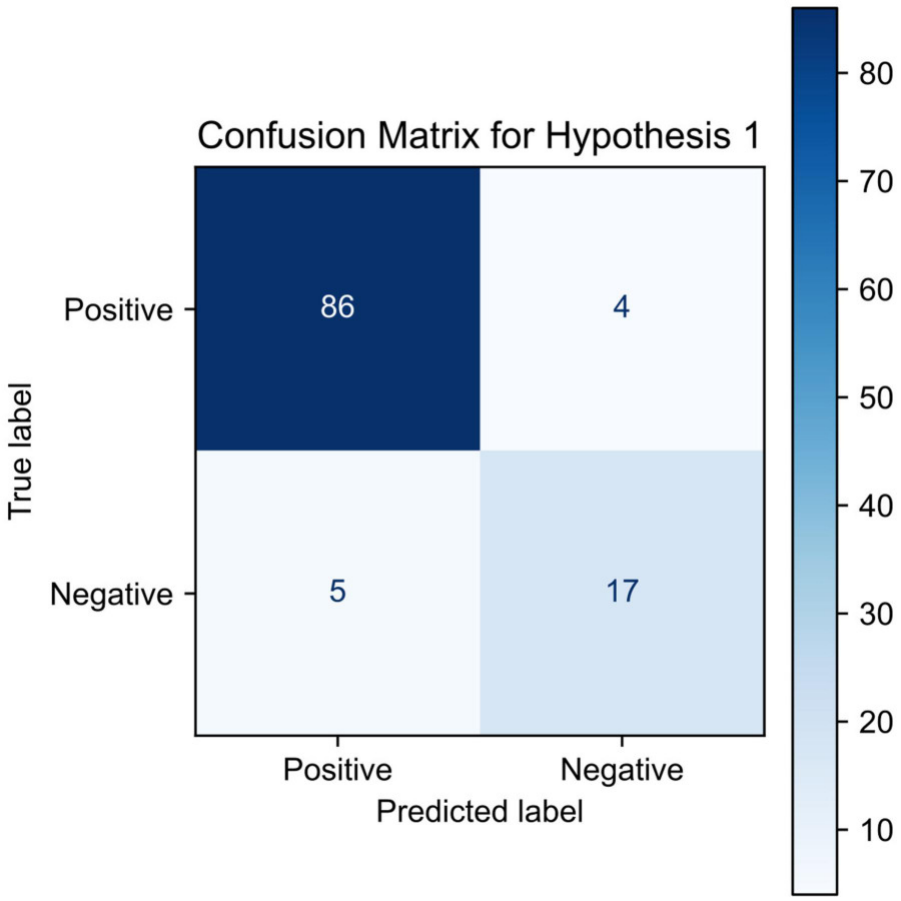
Confusion matrix for Hypothesis 1, illustrating BG responses to non-standard PA. High true positive (TP) and true negative (TN) counts support the hypothesis, confirming that non-standard PA often precedes a BG drop, while standard PA aligns with stable or rising BG trends.

To quantitatively assess the hypothesis when non-standard PA intensifies the BG gradient decline when BG is already falling, we developed confusion matrices across 20, 40, and 60 min windows. This informed our confusion matrix construction, allowing us to systematically categorise and evaluate the predictive performance of non-standard PA on BG gradient behaviour. The confusion matrix thus provided a quantitative assessment of the predictive accuracy of our hypothesis, demonstrating that non-standard PA indeed correlates with intensified BG gradient declines, validating the hypothesis and offering insights into the dynamic interplay between PA and BG levels. Later, we constructed a confusion matrix based on this premise for 20, 40, and 60 min. where Figure 2 is one of the matrices calculated for Hypothesis 1.

In Table 5, we interpret the relationship between PA types and subsequent BG changes in the context of Hypothesis 1 where we say that every non-standard PA, defined as irregular or higher than usual activity, often precedes a BG gradient decline, typically observed in future intervals (x+20, x+40, or x+60) relative to earlier ones (x−20 to x). This is also applicable if the decline is occurring at the same rate. When this expected drop occurs, it is marked as a True Positive (TP) as the change in BG levels has been increased. For every non-standard PA, if the BG gradient continues to drop at the same rate when compared with the prior interval, it is also classified as TP, reflecting that a non-standard PA will keep dropping the BG at the same rate.

**Table 5:** Confusion matrix interpretation for blood glucose (BG) response classification based on physical activity (PA) type in the context of Hypothesis 1.

PA type	Glucose change increase	Glucose change same	Glucose change decrease
Non-standard PA	TP	TP	FP
Standard PA	FN	FN	TN

TP, true positive; FP, false positive; TN, true negative; FN, false negative.

Sometimes, a delayed or buffered physiological response potentially influenced by prior PA, insulin, carbohydrate intake, or individual variability could also be the reason for dropping BG at the same rate. It is well established that the glycaemic effects of PA, particularly increases in insulin sensitivity and muscle glucose uptake, may manifest gradually rather than instantaneously [6, 10]. Consequently, a continued enhanced BG decline following non-standard PA, even without an immediate increase in slope, is most plausibly attributable to activity in preceding intervals rather than a new or corrective physiological response.

A rise in BG gradient during non-standard PA is considered a False Positive (FP). This means that the BG change is starting to decrease compared to the previous interval. In contrast, standard PA is associated with more predictable glycaemic patterns: a rising BG trend is a True Negative (TN), while an unexpected drop under these conditions is marked as a False Negative (FN), as well as when BG change remains the same, as these are not caused due to standard activity. This classification framework supports Hypothesis 1 and is formalised in Equation 1.

(1) $$\begin{array}{rl} & \text{BG}[x]>\text{BG}[x+t],\text{non-standard PA occurs at}\;x,\\ & \text{then}\;\Delta \text{BG}[x-t]<\Delta \;\text{BG}[x+t] \end{array}$$

Hypothesis 2 suggests that if the BG gradient is initially increasing, a decline in the BG gradient is observed after a specific duration when non-standard PA occurs. This implies that following the initial rise, the trend may plateau or reverse, indicating a possible corrective effect influenced by the non-standard PA. Hypothesis 2 formed the foundation for our subsequent analysis and investigations into the temporal relationship between BG gradient behaviour and PA patterns as described in Algorithm 6.

In Table 6, we interpret the relationship between PA types and subsequent changes in the BG gradient in the context of Hypothesis 2. Here, the focus is on cases where the BG gradient initially rises, but a non-standard PA event occurs at time x. Such activity may decrease or reverse the expected increase in the BG gradient, resulting in a smaller rise, or even a decline at future intervals (x+20, x+40, or x+60 min) compared to earlier intervals (x−20 to x). When this expected reversal occurs, it is marked as a TP, as defined in Equation 2. If the BG gradient change remains the same after non-standard PA, meaning if the change is increasing at the same rate when compared with the prior interval change, we classify it as FP. Also, a continued or amplified rise in the BG gradient despite non-standard PA is considered FP. In contrast, when only standard PA, which is compensated by basal rate, is present, and the BG gradient continues rising as expected or continues to rise with the same BG change when compared with the previous interval, the case is labelled a TN. However, if a decline in the BG gradient occurs under standard PA, it is marked as FN.


(2) $$\begin{array}{rl} & \text{BG}[x]<\text{BG}[x+t],\text{non-standard PA occurs at}\;x,\\ & \text{then}\;\Delta \text{BG}[x-t]>\Delta \;\text{BG}[x+t] \end{array}$$

**Table 6:** Confusion matrix interpretation for blood glucose (BG) response classification based on physical activity (PA) type in the context of Hypothesis 2.

PA type	Glucose change increase	Glucose change same	Glucose change decrease
Non-standard PA	FP	FP	TP
Standard PA	TN	TN	FN

TP, true positive; FP, false positive; TN, true negative; FN, false negative.

We reported results at 20, 40, and 60 min windows, which were derived from highly granular raw data streams (15 s PA epochs and 5 min CGM samples). The windowing process was chosen to smooth variability, reflect physiologically meaningful absorption periods, and enable consistent comparison across participants. This proof-of-concept analysis did not measure the extent of BG drops or clinical outcomes, such as the rate of hypoglycemia, Time in Range, Time Above Range, or Time Below Range. Instead, we categorised BG trend responses to non-standard PA into TP, TN, FP, and FN across various time windows to examine temporal associations.

To evaluate the hypotheses, we first computed confusion matrices by comparing predicted BG trends (based on non-standard PA presence) against observed BG outcomes after 20, 40, and 60 min. Each participant’s data were segmented into time windows, and TP, FP, TN, and FN values were derived based on whether the actual post-PA BG trend matched the hypothesis. For instance, in Hypothesis 1, a TP indicates a further BG decline after PA during an already falling BG trend, while an FP would suggest a predicted decline that did not occur. These values were then used to calculate Accuracy, Precision, Sensitivity, and Specificity for each participant per window.

## 3 Results

The study’s results were evaluated based on two hypotheses, analysing the impact of non-standard PA on BG levels in individuals with T1DM. Hypothesis 1 states a further decline in the BG gradient after a specific duration when the BG gradient was already falling, accompanied by non-standard PA. Hypothesis 2 suggested a decrease in the BG gradient after a particular duration when the BG was increasing, accompanied by non-standard PA. The data from eight participants were analysed in 20, 40, and 60 min intervals to test these hypotheses. We tested both hypotheses for all eight participants. This comprehensive approach ensured that both hypotheses were thoroughly evaluated across all eight participants.

The Tables 5, 6 outline the confusion matrix used to classify BG responses to non-standard PA. It includes TP, FP, TN and FN based on BG changes observed before and after PA. The classification aims to evaluate the relationship between non-standard PA and BG fluctuations, assessing the accuracy of the hypotheses. This matrix is crucial for measuring the model’s performance and will provide key metrics such as accuracy, precision, sensitivity, and specificity.

### 3.1 Model performance across participants

The results demonstrate varying accuracy rates, precision, sensitivity, and specificity levels across different time intervals. Participants for both hypotheses in 20, 40, and 60 min windows are defined in Tables 7–9 for Hypothesis 1, and in Tables 10–12 for Hypothesis 2. The percentages in the results are calculated by taking an average of all the values for TP, TN, FP and FN of each individual in 20, 40, and 60 min windows in the confusion matrix with rationale defined in Tables 5, 6.

**Table 7:** Results of Hypothesis 1 at 20 min—Performance metrics for classification of physical activity categories for each participant ID. Accuracy, Precision, Sensitivity, and Specificity are reported as percentages.

ID	Accuracy	Precision	Sensitivity	Specificity
ID01	86.96%	83.82%	98.28%	67.65%
ID02	89.42%	90.59%	96.25%	66.67%
ID03	90.83%	92.59%	94.94%	80.00%
ID04	88.57%	90.48%	90.48%	85.71%
ID05	92.54%	91.80%	91.80%	93.15%
ID06	91.96%	95.56%	94.51%	80.95%
ID07	91.47%	89.77%	97.53%	81.25%
ID08	89.09%	85.00%	94.44%	83.93%

**Table 8:** Results of Hypothesis 1 at 40 min—Accuracy and precision remain consistent, but specificity fluctuates, indicating varying effectiveness in detecting true negative cases.

ID	Accuracy	Precision	Sensitivity	Specificity
ID01	90.00%	85.00%	94.44%	86.36%
ID02	90.91%	90.00%	90.00%	91.67%
ID03	96.08%	96.77%	96.77%	95.00%
ID04	91.49%	87.10%	100.00%	80.00%
ID05	87.88%	85.00%	94.44%	80.00%
ID06	84.48%	89.47%	87.18%	78.95%
ID07	92.73%	96.43%	90.00%	96.00%
ID08	87.76%	91.18%	91.18%	80.00%

**Table 9:** Results of Hypothesis 1 at 60 min—Sensitivity remains high, whereas reduced specificity in certain participants may reflect individual glucose regulation strategies.

ID	Accuracy	Precision	Sensitivity	Specificity
ID01	87.50%	90.91%	83.33%	91.67%
ID02	92.86%	94.44%	94.44%	90.00%
ID03	83.78%	86.67%	92.86%	55.56%
ID04	83.33%	86.67%	81.25%	85.71%
ID05	89.74%	78.57%	91.67%	88.89%
ID06	85.71%	94.74%	81.82%	92.31%
ID07	100.00%	100.00%	100.00%	100.00%
ID08	88.89%	84.62%	91.67%	86.67%

**Table 10:** Results of Hypothesis 2 at 20 min—accuracy and sensitivity are high, confirming the model’s ability to detect glucose drops following prior increases and non-standard activity.

ID	Accuracy	Precision	Sensitivity	Specificity
ID01	87.38%	88.31%	94.44%	70.97%
ID02	81.97%	81.72%	93.83%	58.54%
ID03	86.43%	88.18%	94.17%	64.86%
ID04	81.45%	79.76%	91.78%	66.67%
ID05	82.14%	81.32%	85.06%	79.01%
ID06	83.33%	80.87%	97.89%	55.10%
ID07	78.95%	76.92%	90.91%	62.50%
ID08	85.37%	84.85%	87.50%	83.05%

**Table 11:** Results of Hypothesis 2 at 40 min indicate stable overall accuracy, while inconsistencies in specificity suggest that external factors may be influencing glucose trends.

ID	Accuracy	Precision	Sensitivity	Specificity
ID01	87.76%	88.89%	88.89%	86.36%
ID02	88.33%	94.29%	86.84%	90.91%
ID03	83.08%	83.33%	85.71%	80.00%
ID04	77.59%	85.29%	78.38%	76.19%
ID05	81.01%	81.13%	89.58%	67.74%
ID06	83.87%	82.93%	91.89%	72.00%
ID07	78.75%	72.09%	86.11%	72.73%
ID08	85.00%	83.72%	94.74%	68.18%

**Table 12:** Results of Hypothesis 2 at 60 min reflect strong sensitivity overall, reduced specificity in several participants suggests potential variability in behavioural or physiological responses.

ID	Accuracy	Precision	Sensitivity	Specificity
ID01	86.67%	81.82%	81.82%	89.47%
ID02	73.53%	77.78%	73.68%	73.33%
ID03	86.11%	83.33%	100.00%	54.55%
ID04	75.00%	86.36%	76.00%	72.73%
ID05	85.45%	85.71%	85.71%	85.19%
ID06	85.37%	70.59%	92.31%	82.14%
ID07	83.33%	80.00%	86.96%	80.00%
ID08	87.18%	89.47%	85.00%	89.47%

In the first hypothesis, the accuracy ranged from a minimum of 83.33% for participant ID04 to a maximum of 100% for participant ID07 in a 60 min window Table 9. Moreover, the precision rate varied in the first hypothesis between 85.00% for ID08 and 100.00% for ID07 Tables 7, 9. All participants exhibited higher sensitivity at 20, 40, and 60 min in the first hypothesis with a mean, median and mode value of 92.47%, 93.65%, and 94.44%, respectively, indicating the model’s ability to identify TP instances correctly Tables 7–9. However, for the specificity, participants ID01, ID02 and ID03 experienced a decline at 20 and 60 min in the first hypothesis. Tables 7, 9.

The results from Hypothesis 2 also display variations in accuracy, precision, sensitivity, and specificity across participants and different time intervals Tables 10–12. In the second hypothesis, the accuracy ranged from a minimum of 73.53% for participant ID02 in a 60 min window to a maximum of 88.33% for the same participant in a 40 min window Tables 11, 12. Moreover, the precision rate varied in the second hypothesis between 70.59% for ID06 in a 60 min window and 94.29% for ID02 in a 40 min window Tables 11, 12. Sensitivity ranged from a minimum of 73.68% for participant ID02 in a 60 min window to a maximum of 100% for ID03 in the same-minute window, Table 12, indicating the model’s ability to identify TP instances correctly. However, for the specificity, the lowest rate was observed in the 20 min window for ID02, which is 58.54%; ID06, which is 55.10% and in the 60 min window for ID03, which is 54.55% Tables 10, 12.

For some participants, notably ID02, ID03, and ID06, specificity dropped below 60% in specific time windows. Upon reviewing their individual BG and PA records, we observed that these low specificity values were often linked to real-world behavioural adaptations, such as carbohydrate consumption following PA and insulin suspension during or after PA. These strategies are commonly used to prevent exercise-induced hypoglycaemia. As a result, the expected decline in BG following non-standard PA did not occur, leading the model to classify these as FP. However, these were not necessarily incorrect predictions. They reflected physiological responses that had been intentionally altered by the individual, revealing the complex interaction between prediction and human behaviour.

### 3.2 F1 score evaluation across time windows and hypotheses

**Figure 3:**
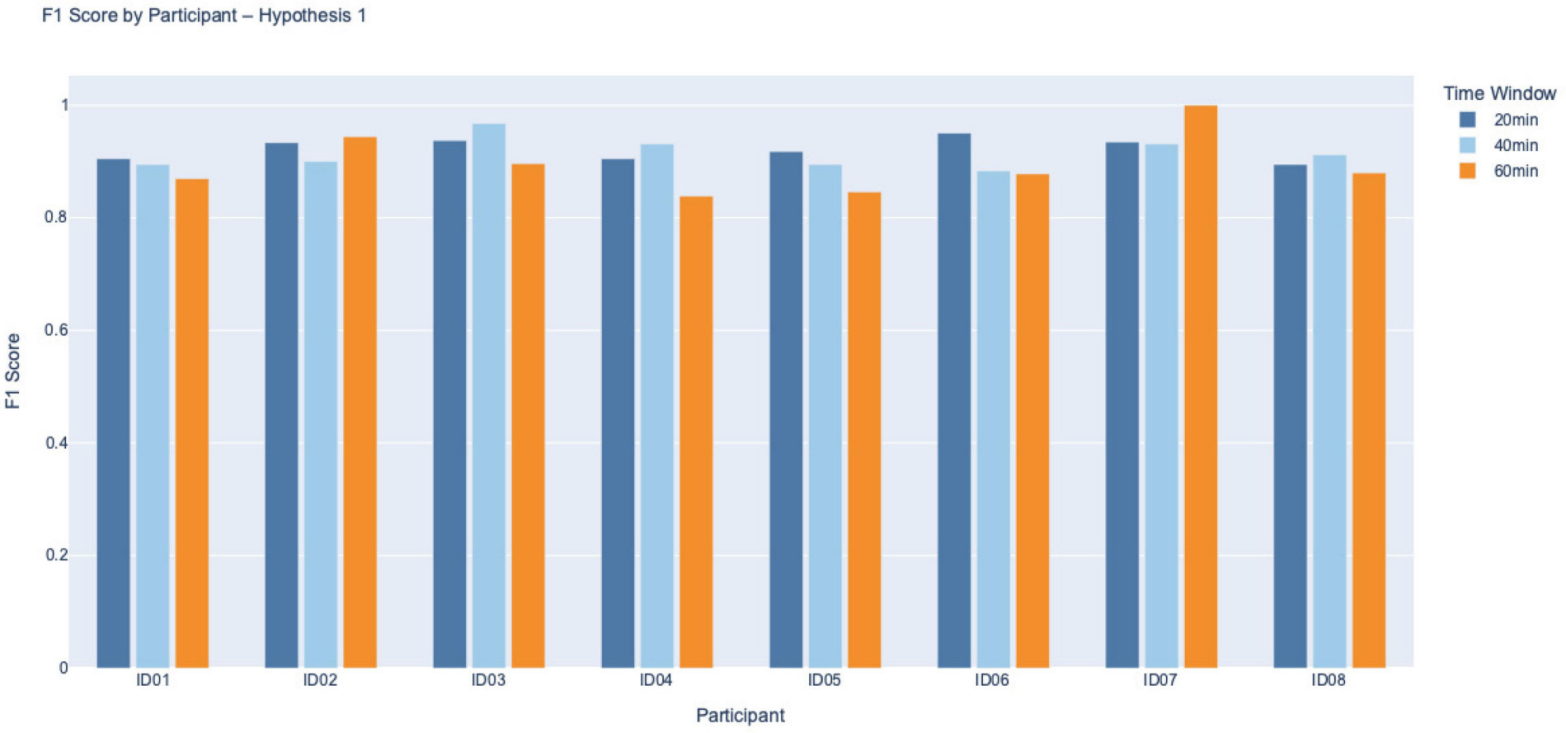
F1 Score by Participant for Hypothesis 1 across 20, 40, and 60 min intervals. Most participants achieved high F1 scores consistently across all time intervals, particularly at 20 min, where several participants, like ID03 and ID07, reached near-perfect performance. This suggests the model’s strong ability to detect glycaemic drops that occur after non-standard PA when BG is already declining, particularly within shorter time frames. A slight decline in F1 was observed with longer windows, indicating reduced predictive accuracy as the interval between PA and BG response increases.

**Figure 4:**
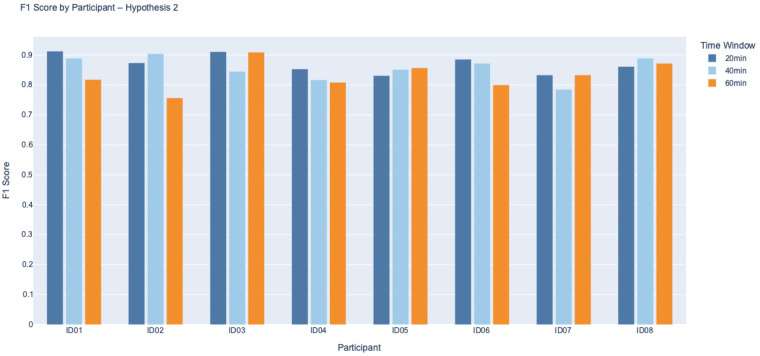
F1 Score by Participant for Hypothesis 2 across 20, 40, and 60 min. While some participants, like ID05 and ID08, maintained strong scores, others, such as ID02 and ID06, indicated substantial reductions, particularly at 60 min. This variability reflects the increased complexity of detecting BG drops following an initial rise, possibly due to delayed or physiological responses. Lower F1 scores at longer intervals highlight reduced classification performance over time in this scenario.

We evaluated model performance using the F1 score, which captures the balance between precision and recall, making it particularly suitable for assessing classification outcomes involving when both FP and FN are of concern. F1 scores were calculated for each participant across both Hypotheses 1, 2, and for three prediction intervals: 20, 40, and 60 min. The FP corresponds to predicting a BG drop that does not actually occur, potentially leading to unnecessary corrective actions (cases where reduced insulin or additional carbohydrate intake) that could lead to hyperglycemia, while an FN represents a missed prediction of an actual BG decline, which could result in unmanaged hypoglycaemia. Figures 3, 4 present the distribution of F1 scores for all eight participants, grouped by hypothesis. Each bar chart displays F1 scores across the three time windows, allowing comparison of classification consistency within and between hypotheses.

Under Hypothesis 1, which suggests a further decline in BG following non-standard PA when BG is already decreasing, the F1 scores were consistently high across participants and time intervals. Most participants achieved F1 scores above 0.85, particularly at the 20 min window. Participants such as ID03 and ID07 approached perfect scores (F1 ≈ 1.0), reflecting strong agreement between predicted and observed BG responses. A slight decline in F1 performance was noted as the prediction interval extended to 60 min, suggesting that the closer temporal proximity between PA and BG change facilitates more accurate classification. It is also important to note that over longer durations, participants often consumed carbohydrates or adjusted insulin to prevent hypoglycaemia, which may have influenced the observed classification performance.

It is important to contextualise these findings in relation to baseline glycaemic control. As shown in Table 1, several participants had baseline HbA1c values above the recommended treatment targets, indicating suboptimal long-term glycaemic regulation. Rather than undermining the results, this heterogeneity reflects the real-world population in which PA-related decision-support tools are most needed. Individuals with higher HbA1c often exhibit greater glycaemic variability and heightened concern regarding hypoglycaemia during PA, which may amplify behavioural responses such as pre-emptive carbohydrate intake or insulin adjustments. Participants with poorer baseline control (ID02 and ID06) showed greater variability and reduced classification performance under Hypothesis 2, particularly at longer prediction horizons. This suggests that baseline metabolic regulation may influence the predictability of BG responses following rising trends, likely through stronger compensatory behaviours or delayed physiological responses.

In contrast, participants with more stable glycaemic profiles (ID05 and ID08) demonstrated more consistent model performance across time windows. This observation aligns with the clinical motivation of the present work. Individuals with suboptimal glycaemic control are often those most reluctant to engage in PA due to fear of unpredictable BG changes. By first establishing that non-standard PA reliably predicts the direction of short-term BG change, even in a cohort with heterogeneous and, in some cases, elevated HbA1c, this framework lays the groundwork for future models that will quantify the magnitude of BG change and support personalised insulin or carbohydrate adjustment recommendations. Such tools have the potential to reduce uncertainty and increase confidence in engaging safely in daily PA, particularly for those with less stable glycaemic control. Overall, F1 scores across both hypotheses demonstrate stronger classification performance at shorter time intervals and during ongoing BG decline. This highlights the method’s effectiveness in detecting immediate glycaemic responses to non-standard PA. In contrast, longer prediction windows, specifically under Hypothesis 2, present greater variability, likely due to individual behaviours such as carbohydrate intake or insulin adjustments during prolonged activity.

### 3.3 Evaluation of hypotheses using the Matthews correlation coefficient

To further validate the reliability of the classification model specifically, when BG is already declining (Hypothesis 1) and when it is rising (Hypothesis 2), we assessed the Matthews correlation coefficient (MCC) in three temporal windows, 20, 40, and 60 min, under two separate hypotheses. MCC was selected for its ability to provide a balanced measure of performance, particularly under conditions of class imbalance, as it incorporates all four outcomes of the confusion matrix (TP, TN, FP and FN). This metric is particularly suitable for binary classification tasks in medical prediction problems where one class may dominate, and precision–recall trade-offs are critical [44].

**Figure 5:**
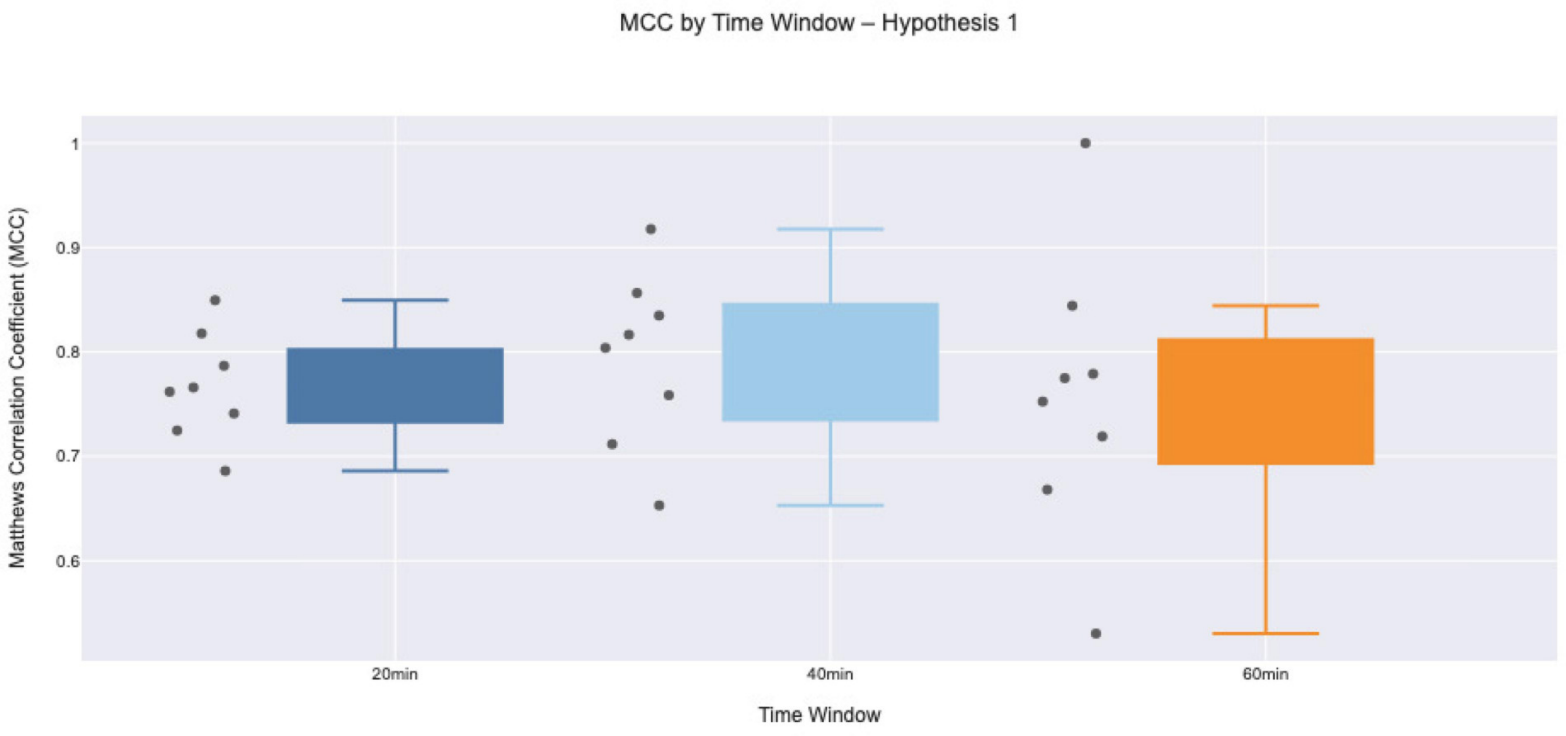
Box plot of Matthews Correlation Coefficient values across participants for Hypothesis 1 at 20, 40, and 60 min intervals. The model shows consistently high performance, with narrow interquartile ranges and few instances of reduced MCC performance.

Under Hypothesis 1, the model achieved consistently high MCC scores in all three time windows Figure 5. The median MCC values remained reliable above 0.75, indicating a reliable agreement between the predicted and observed outcomes. The 40 min window exhibited the highest concentration of elevated scores, with a reduced interquartile range, suggesting low variability between participants in performance. This consistency reflects that the prediction results were primarily driven by the physiological impact of PA, with minimal interference from external behaviour (carbohydrate intake) or treatment-related factors (bolus dosing). Although slightly reduced scores were observed at 60 min due to participant-initiated carbohydrate intake aimed at mitigating the risk of hypoglycaemia during or after PA, overall performance remained within an acceptable predictive range.

**Figure 6:**
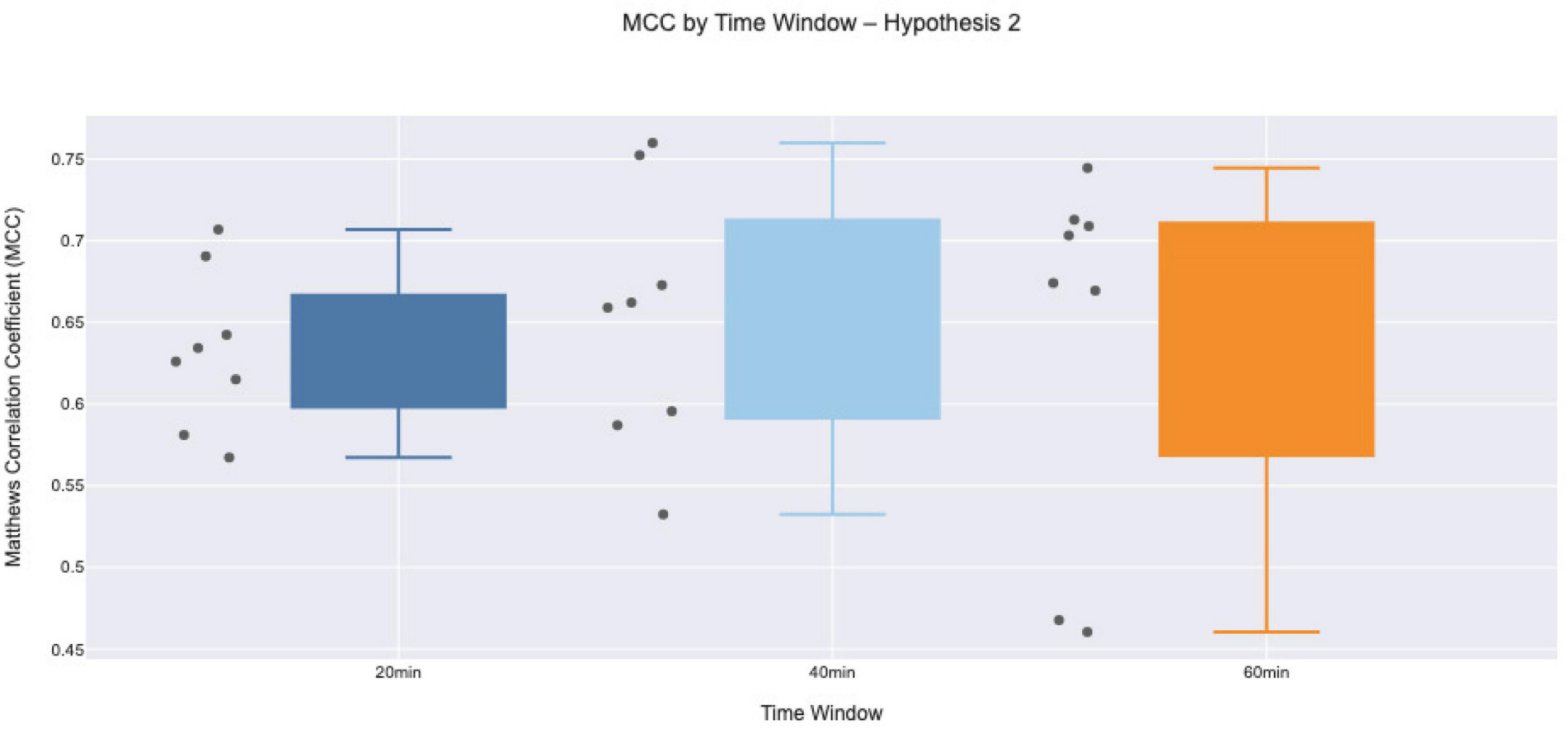
Box plot of Matthews Correlation Coefficient values across participants for Hypothesis 2 at 20, 40, and 60 min intervals. Greater variability and lower medians suggest reduced model reliability, potentially due to behavioural adjustments affecting BG outcomes.

Under Hypothesis 2, where the model indicates BG drops following prior increases, MCC values were generally lower and more variable than in Hypothesis 1. As illustrated in Figure 6, the median MCC values across all three time windows remained between approximately 0.6 and 0.7, indicating moderate predictive performance. However, the interquartile ranges were wider, particularly at the 40 and 60 min intervals, highlighting inconsistencies in the model’s ability to capture BG reversals in the presence of prior rising trends. The drop in specificity for several participants likely contributed to the reduced MCC scores. This suggests that individual counteractive behaviours (carbohydrate intake or insulin adjustments) during rising BG periods might have masked or altered the expected BG drops, likely as part of strategies to prevent hypoglycaemia following PA, introducing more FP and FN. These behaviours are not explicitly captured in the input data, thus reducing the model’s ability to distinguish true physiological declines from regulated trends.

The temporal lag between PA and the corresponding BG response introduces uncertainty, particularly at longer intervals (60 min), where unmeasured factors such as meals, stress, or delayed insulin action may confound outcomes. This likely contributes to the broader variability and lower median MCC values observed at later time windows, particularly under Hypothesis 2. Overall, the model performs more reliably when BG is declining without behavioural intervention (Hypothesis 1), but predictive accuracy declines when prior BG trends are shaped by behavioural interventions (Hypothesis 2). These findings emphasise the promise of PA-based prediction while highlighting the need to incorporate contextual data, such as insulin delivery, carbohydrate intake, and behavioural annotations, to enhance model reliability in real-world applications.

The MCC was computed directly from the confusion matrix counts, TP, TN, FP, and FN, using the standard analytical formulation as shown in Equation 3:

(3) $$\mathrm{MCC}=\frac{\left(\mathrm{TP}\times \mathrm{TN})-(\mathrm{FP}\times \mathrm{FN}\right)}{\sqrt{\left(\mathrm{TP}+\mathrm{FP})(\mathrm{TP}+\mathrm{FN})(\mathrm{TN}+\mathrm{FP})(\mathrm{TN}+\mathrm{FN}\right)}}.$$

MCC values were computed independently for each participant, hypothesis, and temporal window (20, 40, and 60 min). For a given participant and time window, a confusion matrix (TP, TN, FP, FN) was constructed by aggregating event-level classifications across all valid intervals, and the MCC was calculated using the standard formulation. This resulted in one MCC value per participant per time window. For each hypothesis and time window, participant-level MCC values were summarised using descriptive statistics and visualised using box plots.

### 3.4 Aggregated outcomes

For Hypothesis 1, the aggregated confusion matrix as shown in the Table 13 outcomes show a majority of TP and TN relative to false classifications. The high mean TP count indicates that, when BG was already declining, the presence of non-standard PA was frequently followed by a continued or intensified decline in the BG gradient across subsequent intervals. This supports the core premise of Hypothesis 1: that non-standard PA amplifies an ongoing downward BG trend. At the same time, the substantial TN counts suggest that during periods of standard PA, defined as activity compensated by basal insulin, BG gradients generally behaved as expected, remaining stable or rising rather than exhibiting an unexpected decline. The comparatively low FP and FN counts indicate that the algorithm rarely misclassified cases where non-standard PA did not influence BG decline or where standard PA coincided with an unexpected drop in BG. Collectively, these results demonstrate that the proposed classification framework reliably distinguishes between compensated and non-compensated activity when BG is already falling, consistent with Equation 1.

**Table 13:** Aggregated confusion matrix outcomes (mean ± SD) across participants for Hypotheses 1, 2.

Metric	Hypothesis 1 (Mean ± STD)	Hypothesis 2 (Mean ± STD)
TP	36.67 ± 24.25	42.54 ± 27.63
TN	21.71 ± 13.88	24.13 ± 13.09
FP	4.00 ± 2.98	8.92 ± 6.79
FN	2.50 ± 1.67	4.71 ± 2.84

TP, true positive; TN, true negative; FP, false positive; FN, false negative.

To aid interpretation, confusion matrix outcomes (TP, FP, TN, FN) were computed per participant by summing event-level classifications across all analysed intervals. For each hypothesis, counts were pooled across the 20, 40, and 60 min windows, then aggregated across participants and summarised as mean ± standard deviation (STD).

For Hypothesis 2, the aggregated results also show high TP and TN counts, but with increased FP and FN values compared to Hypothesis 1. The elevated TP counts indicate that, when BG was initially rising, non-standard PA often preceded a reduction in the rate of increase or a subsequent decline in the BG gradient, consistent with the hypothesised delayed or corrective effect of PA. This supports the notion that non-standard PA can counteract an upward BG trajectory, even when the immediate trend is increasing. However, the higher FP and FN counts reflect greater physiological variability in this context. In some cases, BG continued to rise despite non-standard PA, likely due to competing influences such as recent carbohydrate intake, insulin on board, or delayed metabolic responses. Nonetheless, the persistence of high TN counts under standard PA conditions indicates that rising BG trends during habitual activity generally remained predictably consistent with Equation 2.

To assess whether the aggregated confusion matrix outcomes reflected a consistent signal across individuals rather than random variation, we performed non-parametric statistical analyses across participants. For each participant, correct classifications (TP + TN) and incorrect classifications (FP + FN) were computed by pooling results across the 20, 40, and 60 min windows, yielding a paired comparison per individual. A Wilcoxon signed-rank test [45] demonstrated that correct classifications significantly exceeded incorrect classifications across all eight participants (*W* = 0, *p* = 0.0078), indicating perfect directional consistency with no contradictory cases. The corresponding rank-biserial correlation (*r* = 1.0) reflects a maximal effect size, confirming that the observed signal was uniformly present across participants.

As a secondary reliabilty check, a Mann–Whitney *U* test [46] was conducted comparing the distributions of correct and incorrect classifications while ignoring participant pairing. This analysis yielded *U* = 64 (maximum possible value), *p* = 0.0005, indicating complete separation between correct and incorrect classifications. Although this test does not account for within-participant pairing and is therefore conceptually weaker for the present design, it provides convergent evidence that the observed classification performance is highly unlikely to be attributable to chance.

Collectively, these results demonstrate that the aggregated confusion matrix outcomes are not driven by a small subset of individuals, but instead reflect a strong and consistent within-participant signal across the entire cohort.

### 3.5 Results analysis

We developed our first hypothesis in Algorithm 5. It suggests that if there is a decrease in BG levels every time a non-standard PA occurs at an *x* interval, then there will be a further decline in the BG gradient compared to the previous time window (x−20). The results were calculated based on the confusion matrix as mentioned in Tables 5, 6. We calculated accuracy, precision, sensitivity, and specificity based on this hypothesis. In the first hypothesis for a 20, 40, and 60 min window, we found the mean accuracy to be 89.75%, precision 89.88%, sensitivity 92.47%, and specificity 84.09%. Based on these results, our model exhibited strong accuracy, precision and sensitivity in Hypothesis 1, correctly identifying positive instances in participants’ BG levels. However, for the specificity, participants ID01, ID02 and ID03 experienced a decline at 20 and 60 min in the first hypothesis. These were not necessarily incorrect predictions but were influenced by behavioural interventions. Upon reviewing the original data of those individuals, we found that they had consumed carbohydrates, the insulin dosage was stopped, or the PA had ceased, which helped to treat their hypoglycaemia by adjusting their BG levels. This aligns with our vision of encouraging participants to consume certain carbohydrates or adjust the insulin dosages to regulate their BG levels for particular PA.

In the second hypothesis for a 20, 40, and 60 min window, we found the mean accuracy to be 83.13%, precision 82.86%, sensitivity 86.48%, and specificity 78.36%. Based on these results, our model exhibited strong accuracy, precision and sensitivity in Hypothesis 2, correctly identifying positive instances in participants’ BG levels. However, the fluctuations in the specificity were observed, the lowest rate was observed in the 20 min window for ID02, which is 58.54%; ID06, which is 55.10% and in the 60 min window for ID03, which is 54.55%. Upon reviewing data of specific individuals, the predictions were not incorrect but rather influenced by behavioural interventions. We discovered that these individuals had consumed carbohydrates or stopped taking insulin to adjust their BG levels when they preferred staying at higher BG levels for a specific time while involved in PA. Overall, these results underscore the need for personalised approaches in predicting BG levels, considering individual variations in PA responses.

Based on our results and analyses, we confirmed a relationship between non-standard PA and BG levels. Our findings indicate that a decrease in BG is observed after non-standard PA, and this decrease becomes more pronounced in the subsequent period. These findings support the hypothesis and motivate the next phase of our research. In our following paper, we will evaluate the effects of different levels of non-standardised PA on BG changes. This will allow us to build a prediction model that estimates BG changes depending on the intensity of non-standard activities, thus increasing our ability to control and predict BG changes effectively.

To ensure the consistency and reliability of our results, we conducted intra-rater reliability testing. It involved the independent examination of a subset of the raw data by another expert in the field. Specifically, 25% of the total dataset, comprising 192 total results, was randomly selected for this assessment. The independent rater reviewed the chosen points and cross-verified each value against the original records. All the data points assessed in this subset were confirmed to be accurate, highlighting consistency and reliability in the measurements.

## 4 Discussions

Despite prior efforts, numerous limitations, such as PA measurement mechanism, PA classification, prediction horizon, and accuracy, must be addressed to improve BG forecasting techniques in T1DM. The PA measurement mechanism encompasses devices like smartwatches or platforms equipped with sensors to gather data on movement and physiological signals. On the other hand, PA classification involves interpreting this data, categorising activities like walking or running, and quantifying metrics such as steps or heart rate. These mechanisms and classifications provide insights into daily activities and overall health. Furthermore, the prediction model should encompass the impact of daily PA rather than solely focusing on the effects of physical exercise on BG levels.

The primary contribution of this paper to the field of T1DM glucose prediction algorithms lies in introducing a classification system for PA: “standard” and “non-standard” PA. In traditional diabetes management, standard PA, such as routine daily tasks, are typically well-compensated by basal insulin rates, which are set to maintain BG levels during periods of minimal PA. However, non-standard PA, which includes more intense or unpredictable physical exertion, present a unique challenge in BG regulation [2, 47, 48]. These activities often cause significant fluctuations in BG levels that cannot be adequately managed by basal insulin alone.

This study is a secondary analysis of data from a previously published trial [36], and eight individuals had complete PA and BG data available for analysis. Importantly, each participant contributed detailed data over five consecutive days, which provided sufficient density to test the method. For an adaptive system, the richness of the dataset is more critical than the number of participants. Across the observation period, we obtained raw PA data at 15 s intervals (≈28,800 points over 5 days) and BG data at 5 min intervals (≈1,440 points over 5 days), representing a large volume of paired data for daytime model development.

The proposed framework is evaluated in a longitudinal, within-subject setting, where inferential strength is primarily driven by the density, consistency, and directionality of repeated observations within individuals, rather than by the absolute number of participants. Each participant contributed several days of high-frequency PA and CGM data, yielding hundreds to thousands of PA–BG interaction events per individual. This design is well suited to adaptive and personalised modelling approaches, in which model behaviour and hypothesis validation emerge from repeated within-person patterns rather than between-person comparisons.

Importantly, once data collection is complete, the relevant question is not how many participants would have been required under a priori assumptions of uncertain effects, but whether the observed signal is sufficiently strong and consistent to be distinguishable from random variation. In this study, non-parametric analyses demonstrated that correct classifications significantly exceeded incorrect classifications across all participants, with perfect directional consistency and large effect sizes. If the underlying physiological association between non-standard PA and subsequent BG gradient behaviour were weak or unstable, it would be unlikely to manifest so clearly in a cohort of only eight individuals. The fact that the signal is detectable with strong statistical support in a small sample therefore indicates a reliable within-participant effect rather than a marginal or noise-driven phenomenon.

We intentionally isolated PA as the sole input to the model in order to attribute observed BG changes directly to activity, rather than to meals or insulin dosing, which are often incompletely or imprecisely recorded in free-living datasets. In real-world conditions, carbohydrate intake is frequently under-reported, inaccurately estimated, or inconsistently timestamped, while insulin records may omit manual boluses, reflect delayed corrections, or fail to capture behavioural intent. Incorporating such noisy and temporally misaligned data alongside high-resolution CGM and wearable PA signals risks introducing substantial measurement error and obscuring the independent glycaemic effect of PA.

Moderate-to-vigorous PA can exert both immediate and delayed effects on BG regulation, including prolonged increases in insulin sensitivity and delayed hypoglycaemia occurring several hours after activity, such as overnight following evening exercise [49]. To address this, our analysis explicitly focused on short-term temporal windows (20, 40, and 60 min) surrounding detected non-standard PA events, where causal attribution between activity and subsequent changes in the BG gradient is most consistent with known PA–BG physiology. By identifying risk patterns close to the time of activity, this framework aims to support future decision-support systems that can provide early recommendations, such as adjustments to carbohydrate intake or insulin, at or shortly after activity, thereby reducing the likelihood of delayed hypoglycaemia later in the day or overnight. Constraining the temporal scope in this way allows us to capture acute and near-term PA-related glycaemic responses while minimising confounding from longer-latency metabolic processes that are influenced by multiple unobserved factors.

Importantly, the observed FP and FN, particularly under Hypothesis 2, are likely to reflect intentional behavioural adaptations rather than model failure. Participants often consumed carbohydrates or modified insulin delivery in anticipation of PA-induced hypoglycaemia, thereby reducing or delaying expected BG responses. In such cases, the algorithm correctly detected non-standard PA, but the physiological outcome was altered by unobserved compensatory actions. As a result, the reported F1-score and MCC are intentionally cautious. Since the model did not know when participants ate carbohydrates or adjusted insulin, some apparent errors were caused by human intervention rather than model failure. The reported metrics therefore, represent a minimum estimate of performance

Rather than viewing the exclusion of insulin and meal data as a purely methodological constraint, this design choice reflects a realistic deployment scenario for PA-aware decision-support systems, where reliable activity and CGM data are consistently available, but detailed behavioural logs are not. Future work will extend this framework by integrating timestamped insulin and dietary inputs to explicitly model delayed PA effects and improve specificity, particularly for delayed hypoglycaemic events. Nonetheless, the present results demonstrate that even in the absence of these inputs, non-standard PA alone provides clinically meaningful information about short-term BG risk under free-living conditions.

In previous studies, researchers have demonstrated increased accuracy in BG prediction by implementing specific exercises [33, 50]. Studies exist to predict BG based on PA and physical exercise in controlled environments [13, 51, 52] in clinical trials [53–55] and free-living conditions [56–58]. Moreover, studies have explored the prediction of BG levels based on PA in silico [59, 60]. Nevertheless, no study has taken the influence of PA on BG considering activities throughout the day, encompassing periods of rest and PA under normal, free-living conditions. Regardless, previous studies were limited to physical exercise conducted at predetermined intervals and did not encompass routine daily activities, whether resting, walking, or doing more physical exercise such as brisk walking or any other spontaneous activities [50]. Our study is situated within the framework of routine daily activities, with participants engaged in various daily tasks.

In the context of ML models, a pivotal consideration revolves around the requirement for extensive and high-quality datasets during the training process [61]. If the data used for training is marred by issues such as incompleteness, bias, or a lack of representativeness mirroring real-world situations, then the struggle to generalise effectively to novel, unseen data instances. This challenge is pronounced in scenarios like T1DM, where missing or invalid data at specific time points significantly compromises the model’s efficiency, leading to less optimal results. In our findings, several intriguing patterns emerged. In instances where inaccuracies occurred in predicting specificity rates, further scrutiny reveals a direct correlation between these inaccuracies and specific participant actions. Specifically, in Hypothesis 2, participants ID02, ID03, and ID06 injected bolus insulin.

In this work, two hypotheses were formulated corresponding to these distinct scenarios based on these observations. Under Hypothesis 1, we proposed stabilising BG levels at a certain level *x* mmol/L following carbohydrate consumption. Applying this hypothesis-driven algorithm, we achieved an accuracy rate of 83.33% to 100% across 20, 40, and 60 min intervals in Hypothesis 1. Similarly, in Hypothesis 2, we postulated that administering insulin would effectively manage BG levels when BG reached a certain level *x* mmol/L (with participant-specific thresholds). Our predictions aligned with this hypothesis, yielding a prediction accuracy rate ranging from 73.53% to 88.33% in these instances. The strength of our research demonstrates a hypothesis-driven algorithm with high accuracy rates, highlighting the effectiveness of targeted interventions in managing BG levels. Another strength is that by studying free-living situations and considering different levels of PA throughout the day, our research captures a more realistic scenario, making the findings applicable to daily life activities for individuals with T1DM.

After analysing discrepancies in Hypotheses 1, 2, we identified that participants often consumed carbohydrates or administered insulin around the time of PA, which directly influenced BG trajectories. These interventions, though necessary for safe diabetes management, disrupted the expected glycaemic patterns, such as the decline anticipated in Hypothesis 1 or the plateau/drop expected in Hypothesis 2, which were not consistently observed at 20, 40, or 60 min. Such variability reflects real-world complexity and reinforces the importance of contextualising physiological trends with behavioural data. The position statement [4] supports proactive BG management strategies during PA. While our current model focuses on PA-induced trends, incorporating time-stamped insulin and carbohydrate events could improve alignment with observed BG responses.

In addition, specificity fell below 60% for several participants, including ID02, ID03, and ID06, under Hypothesis 2, particularly during episodes involving non-standard PA. On closer inspection, these cases often coincided with safety-driven behaviours, such as temporary insulin pump suspension or rapid-acting carbohydrate intake performed in anticipation of, or response to, perceived hypoglycaemic risk. These decisions, although clinically prudent, reduced the model’s ability to detect the isolated effect of PA on delayed BG changes. Furthermore, while PA intensity in this study was defined as activity counts surpassing each participant’s daily average (non-standard PA), future research will categorise non-standard PA into quartiles: low, medium, high, and very high. This approach will permit an assessment of the glycaemic impact for each specific level of PA intensity, improving clinical understanding and facilitating more personalised recommendations for insulin or carbohydrate adjustments.

The objective of this study was to validate the temporal and directional structure linking non-standard PA to subsequent BG trends under free-living conditions. Having established that non-standard PA reliably predicts the direction of BG change, now we have the foundations to focus on estimating the magnitude of BG response across various classified PA intensity levels (low, medium, high, and very high). This reflects standard model development practice, in which the relationship between variables is first validated before estimating effect magnitude and translating results into clinical decision support, such as deriving personalised insulin or carbohydrate adjustment recommendations. The confirmation of these hypotheses, therefore, represents a necessary first step toward quantitative modelling.

### 4.1 Limitations

Participants had advanced age and long duration of diabetes among participants, which may be associated with reduced cardiovascular fitness, muscle strength, and joint flexibility, thereby limiting PA capacity. However, the adaptive framework was designed at the individual level, using each participant’s own mean PA as the reference rather than a population-wide average. This ensures that predictions reflect personal activity patterns, even within an older cohort. Each participant provided detailed, continuous data over multiple days, generating a large number of paired PA and BG points. For an adaptive, within-person framework such as ours, the richness of the dataset is more critical than the overall sample size.

## 5 Conclusion and future work

In this study, we evaluated a temporal gradient framework to assess how non-standard PA influences the direction and rate of BG changes in individuals with T1DM. Our findings revealed consistent temporal associations, indicating that non-standard PA often preceded steeper declines in BG levels. This framework could improve the accuracy of insulin and carbohydrate recommendations in relation to PA. However, these results should be considered a preliminary proof-of-concept and are not yet generalisable or clinically actionable. Future research will focus on quantifying the magnitude of BG changes based on PA intensity, leading to the development of a predictive model that estimates expected BG decline across varying levels of PA. Such precision is essential for optimising insulin dosing and carbohydrate intake, ultimately minimising the risk of hypoglycaemia.

## Data Availability

The original contributions presented in the study are publicly available. This data can be found here: https://doi.org/10.5281/zenodo.17991897.

## Appendix

Algorithm 1 Handling Active States of Activity in Dataset.

Algorithm 2 Resampling and Aggregation of Activity and Glucose Data.

Algorithm 3 Merging Activity and Glucose Data.

Algorithm 4 Calculate Activity and Glucose Gradients.

Algorithm 5 Hypothesis 1 - Detection of Increased BG Drop Following Non-Standard Activity.

Algorithm 6 Hypothesis 2 - Detection of BG Decline After Initial Rise and Non-Standard Activity.
